# Recognizing and validating ligands with CheckMyBlob

**DOI:** 10.1093/nar/gkab296

**Published:** 2021-04-27

**Authors:** Dariusz Brzezinski, Przemyslaw J Porebski, Marcin Kowiel, Joanna M Macnar, Wladek Minor

**Affiliations:** Department of Molecular Physiology and Biological Physics, University of Virginia, Charlottesville, VA 22908, USA; Institute of Computing Science, Poznan University of Technology, Poznan, 60-965, Poland; Center for Biocrystallographic Research, Institute of Bioorganic Chemistry, Polish Academy of Sciences, Poznan, 61-704, Poland; Department of Molecular Physiology and Biological Physics, University of Virginia, Charlottesville, VA 22908, USA; Center for Biocrystallographic Research, Institute of Bioorganic Chemistry, Polish Academy of Sciences, Poznan, 61-704, Poland; Department of Molecular Physiology and Biological Physics, University of Virginia, Charlottesville, VA 22908, USA; College of Inter-Faculty Individual Studies in Mathematics and Natural Sciences, University of Warsaw, Warsaw, 02-097, Poland; Faculty of Chemistry, Biological and Chemical Research Center, University of Warsaw, Warsaw, 02-089, Poland; Department of Molecular Physiology and Biological Physics, University of Virginia, Charlottesville, VA 22908, USA

## Abstract

Structure-guided drug design depends on the correct identification of ligands in crystal structures of protein complexes. However, the interpretation of the electron density maps is challenging and often burdened with confirmation bias. Ligand identification can be aided by automatic methods such as CheckMyBlob, a machine learning algorithm that learns to generalize ligand descriptions from sets of moieties deposited in the Protein Data Bank. Here, we present the CheckMyBlob web server, a platform that can identify ligands in unmodeled fragments of electron density maps or validate ligands in existing models. The server processes PDB/mmCIF and MTZ files and returns a ranking of 10 most likely ligands for each detected electron density blob along with interactive 3D visualizations. Additionally, for each prediction/validation, a plugin script is generated that enables users to conduct a detailed analysis of the server results in Coot. The CheckMyBlob web server is available at https://checkmyblob.bioreproducibility.org.

## Graphical Abstract

**Graphical Abstract:**
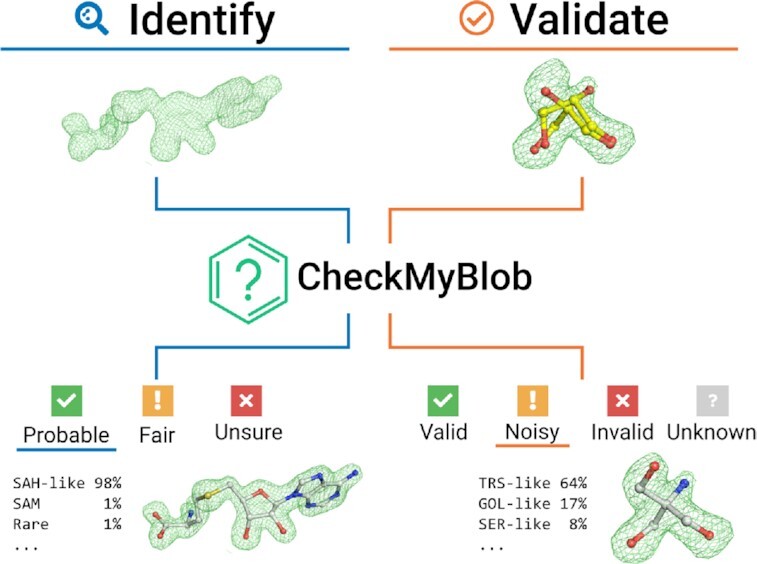
CheckMyBlob is a machine learning system that automatically detects and validates ligands in X-ray electron density maps.

## INTRODUCTION

Many macromolecular crystal structures contain ligand molecules that can reveal the function of the protein or nucleic acid. Ligands are usually manually modeled by crystallographers, which requires good judgment and expertise. This process is time-consuming and prone to human error, particularly when the resolution of the diffraction data is not very high, there is local disorder, or the ligand is bound to only a fraction of the molecules. The often-questionable assignment of ligands to electron density ‘blobs’ (1) shows that automatic methods for ligand recognition and validation are needed to streamline the interpretation and remove potential cognitive bias (2).

Several approaches are used to automate fitting *known* ligands to electron density maps. They are typically based on recognition of the ligand's core atoms followed by iterative element addition (3,4), Metropolis-type optimization (5), or similar techniques (6–8). These methods can be adapted to identify *unknown* ligands by iteratively fitting moieties from a predefined list of candidates, an approach (9) that was reported to achieve 48% accuracy in recognizing instances of the 200 most frequently observed ligands in structures stored in the Protein Data Bank (PDB) (10). However, for thorough large-scale searches evaluating tens or hundreds of ligands, these approaches can be prohibitively slow. Alternatives to iterative fitting mainly use mathematical descriptions of 3D electron density map fragments (11–15). Although these methods are much faster than iterative ligand fitting, the best approaches from this group only achieve 30–32% accuracy in identifying the correct ligand from a list of the 100 most common ligands (13,15).

CheckMyBlob (16) is a machine learning based algorithm that identifies ligands in electron density maps. In contrast to the methods mentioned above, CheckMyBlob learns to generalize ligand descriptions from sets of moieties deposited in the PDB, rather than comparing density maps to theoretical models, graphs, or selected template structures. Moreover, in contrast to other methods, the algorithm does not require human intervention during the ligand recognition process, making it a completely automatic approach for the initial interpretation of residual electron density blobs after structure determination and preliminary biopolymer refinement. In benchmark tests on portfolios of up to 219 931 ligands, CheckMyBlob achieved 73% accuracy in recognizing instances of the 100 most popular ligands and 56% accuracy in recognizing the 200 most popular ligands, while requiring significantly less time than competitive approaches (16).

Here, we present the CheckMyBlob web server, a tool that helps users identify ligands in unmodeled fragments of electron density maps or validate ligands in existing models. The web server uses the CheckMyBlob algorithm trained to recognize over 200 classes of ligands grouped according to their geometry and molecular weight. Moreover, the web server model was trained to recognize metal ions, water molecules, and polymers (ligands that the PDB encodes as several monomers). The server visualizes the detected electron density blobs, and for each prediction/validation, a plugin script is generated that enables users to analyze the results in Coot (17). To the best of our knowledge, this is the first online tool to automatically identify and predict ligands in electron density maps.

## MATERIALS AND METHODS

### Data collection and curation

To obtain training data for the ligand recognition model, we downloaded all PDB entries as of 19 January 2020. We converted the associated structure factors from mmCIF to mtz format using the cif2mtz program from the CCP4 suite (18). Due to the large number of water molecules in macromolecular structures, only a subset was added to the training data. In an attempt to sample water molecules from a diverse set of structures, we divided the pool of available PDB deposits into 25 evenly-populated resolution bins. Next, within each bin we chose deposits that were non-redundant at a 50% pairwise sequence identity threshold, as calculated by BLASTclust (19). Therefore, to sample water molecules we chose structures of proteins that were significantly different from each other. This procedure resulted in 241 709 water molecule blobs extracted from 10 726 structures, which were later filtered according to various quality criteria (see below). In total, the data collection process resulted in 957 855 blob descriptions.

As input to the ligand identification pipeline, we calculated *F*_o_ *– F*_c_ electron density maps, with 0.2 Å grid spacing, based on data in the mtz files and on atomic coordinates of the main-chain and side-chain atoms. Small-molecule moieties and solvent molecules were excluded. The resulting partial models were refined with five cycles of REFMAC (20) to reduce the ‘memory’ and modeling bias of the excluded molecules in the calculated structure factors and maps. Next, blobs were automatically found by analyzing all positive electron density peaks within the *F*_o_*– F*_c_ map. To mitigate the problem of ligands being divided into multiple blobs, the system detected local maxima, skeletonized the electron density within each blob's isosurface, and combined adjacent blobs when the distance between the local maxima or skeleton nodes was less than 2.15 Å (16). Any fragments of electron density in the blob isosurface that overlapped with the isosurface of the modeled biopolymer atoms were cut out from the blob. Finally, each detected ligand was described by a set of numerical features, such as 3D shape descriptors, blob volume, map statistics, and PCA eigenvalues based on positive peaks from *F*_o_*– F*_c_ maps. A detailed description of the data processing, ligand detection, and ligand label assignment methods can be found in (16).

The final training data set consisted of ligands from X-ray diffraction experiments of at least 4.0 Å resolution. We also eliminated all suspicious deposits and ligands according to various quality criteria, such as RSCC < 0.6, real space *Z*_obs_ (RSZO) < 1.0, real space *Z*_diff_ (RSZD) ≥6.0, R factor >0.3, or occupancy <0.3. The details of the filtering process are described in (16). The resulting training data set consisted of 696 887 examples of blobs, which were assigned ligand labels (clustered).

### Ligand clustering

Several ligands are indistinguishable by electron density alone. For example, for most resolution ranges, it is extremely challenging to distinguish between 6, 7 or 8 electrons (carbon, nitrogen, oxygen) or distinguish between single, double, or triple bonds in some configurations. To improve the robustness of the classification algorithm, we decided to cluster ligands based on the expected shape of their electron density derived from their chemical definitions. Namely, we considered the atom connectivity, chirality and significant differences in the number of electrons but disregarded atom types and bond order in some equivalent bond configurations. The clustering procedure was as follows:

1. Group molecules by matching number of atoms, rings, and aromatic rings;
2. Group by connectivity (matching substructures using generic atoms and generic bonds);
3. Check if the chirality of all matching combinations agrees;
4. Check if the following SMART patterns match in equivalent positions: [!D1]#[!D1] (non-terminal triple bond), [!D1]=*=[!D1] (subsequent non-terminal double bonds), [D1]#[D2]-* or [D1]=[D2]=* (linear configuration of three atoms);
5. Check if the equivalent atoms are in the same atomic number group (<5, 5–13, 14–20, 21–31, 32–38, 39–49, >50).

**Figure 1.**
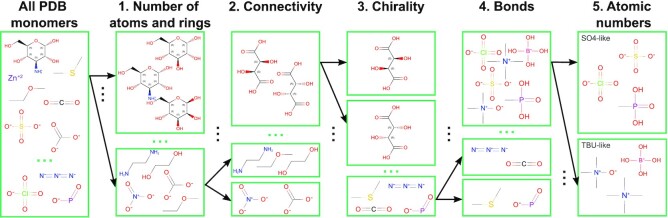
Schematic of the ligand clustering procedure. Arrows depict example cluster splits based on different criteria at subsequent stages of the process.

A schematic of the clustering procedure is presented in Figure 1.

Clustering was performed using RDKit with PostgreSQL and Python interfaces (https://www.rdkit.org). Chemical descriptions of ligands were based on the SMILES and InChI descriptors provided by the PDB.

### Classification model

The primary classification model was trained to recognize the 219 most popular ligand groups (clusters). This number was achieved by limiting the training to classes with at least 100 examples in the training set. All the ligands that were not in those 219 groups were labeled as a separate class called *rare*. When the primary classification model predicts *rare*, the example is additionally processed by a secondary model trained only on ligands in the *rare* group.

The server's primary classification model was trained using the gradient boosting machine (GBM) algorithm (21). The classifier used the same features and hyperparameters as described in (16). To preprocess the data, the server uses the scikit-learn library v.0.20.4, whereas the GBM implementation was taken from Microsoft's LightGBM package v.2.3.0. The secondary model that provides predictions for the class *rare* was a 1-NN classifier (22). By using a 1-NN classifier as a secondary model, even ligands that occur only once in the PDB have a chance of being predicted.

### Model evaluation results

The resulting machine learning model was first validated in a stratified 10-fold cross-validation experiment involving the gathered 696 887 training ligand instances with a mean resolution of 2.2 Å. The model achieved 71% accuracy and 95% top-10 accuracy (the chance that the correct ligand group is within the server's suggestion of 10 ligands). After the model was created, we processed additional (held out) PDB deposits with the same pipeline and created a separate test set. The test set included 17 150 ligand instances with a mean resolution of 2.5 Å, on which the model achieved 59% accuracy and 93% top-10 accuracy. The holdout test set was more challenging because it contained lower resolution structures, but the results are still superior to those reported for non-clustered ligands with at least two non-hydrogen atoms (16). A summary of the model's performance, according to different evaluation measures (23), is presented in Table 1. The ‘About’ page of the CheckMyBlob web server contains an interactive calibration plot and a confusion matrix that describes the predictive performance for particular ligands.

**Table 1.** Basic statistics and average predictive performance metrics with standard deviations (in parentheses, in the unit of the last significant digit of the mean value) on the cross-validated (10-fold CV) training set and the holdout test set

	10-fold CV	Holdout set
Ligand instances	696 887	17 150
Mean resolution (Å)	2.2	2.5
Accuracy (%)	71.2(9)	58.9
Top-5 accuracy (%)	90.7(5)	87.2
Top-10 accuracy (%)	94.9(2)	92.5
Micro-averaged recall (%)	71.2(9)	58.9
Micro-averaged precision (%)	69.3(11)	62.7
Micro-averaged F1 (%)	69.3(11)	55.7
Cohen's kappa (%)	64.6(12)	46.2

### Server implementation

The web server was developed in Python using the Django framework (https://www.djangoproject.com) with a PostgreSQL database (https://www.postgresql.org) to store ligand definitions and job processing data. The submitted jobs are queued using Celery (https://docs.celeryproject.org/) with a RabbitMQ message broker (https://www.rabbitmq.com/). All the website pages are formatted with Bootstrap (https://getbootstrap.com), and the result views are dynamically created using the doT templating engine (https://olado.github.io/). The website also uses the NGL JavaScript library for electron density visualization (24,25).

## RESULTS

### Workflow

**Figure 2.**
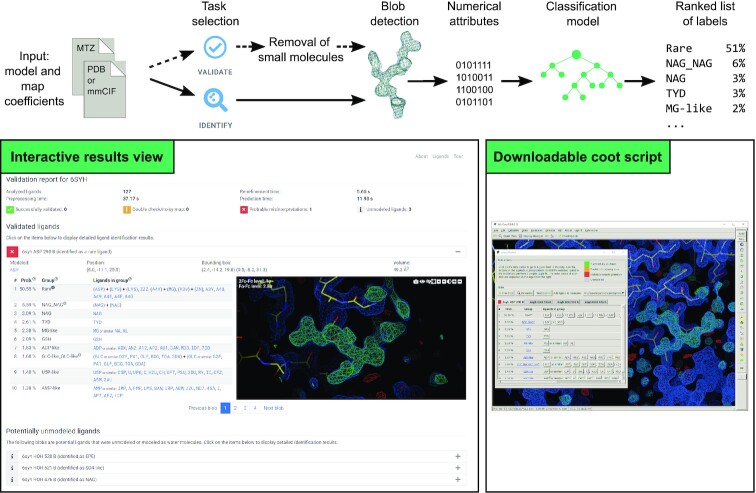
Schematic representation of the CheckMyBlob workflow and screenshots of the interactive results visualization page and Coot ligand analysis script. The user provides input files (an MTZ file and PDB or mmCIF file) and chooses to either detect unmodeled ligands or validate existing ligands. Next, blobs are detected, extracted from electron density maps, and described by a set of numerical features. The obtained numerical features are input to a machine learning model, which outputs a ranking of the ten most likely ligands for each blob. This probability-based ranking can be viewed on the interactive results visualization page and tested in Coot through a downloadable script.

The CheckMyBlob protocol is presented as a workflow in Figure 2.

#### Input

Users can choose between two tasks that the server can perform: 1) the *Identify* task, where the server looks for unmodeled ligands and 2) the *Validate* task, where the server compares existing ligands with CheckMyBlob's predictions. To perform these tasks, users must submit a structural model in the form of a PDB or mmCIF file and MTZ map coefficients. In the *Validate* task, users can also provide the PDB code of an existing deposit. In both cases, the user can optionally choose to evaluate water molecules, which can be used to verify whether a set of water molecules was inserted instead of a potential ligand.

#### Processing and prediction

The input files are first prepared for blob detection. In the *Identify* task, this means running a zero-cycle of refinement and in the *Validate* task this means removing all the small-molecules from the model and running five cycles of refinement. Following data preparation, the blobs are detected, described by numerical features, and fed to the classification model. In the identification task, the classifier is a model trained on the entire PDB. In contrast, during the validation of an existing PDB deposit, the server uses a model that was trained on 90% of the PDB, excluding the deposit in question. Therefore, the validation of existing PDB deposits is always done using a model that was not trained on the structure being validated. Following model inference, a ranking of ten ligand predictions is prepared. If the primary classification model predicts the *rare* class within the top 10 most probable ligand groups, the secondary model is used to predict a set of 10 rare ligands.

#### Result presentation

CheckMyBlob predictions are displayed as an interactive report (Figure 2). Each detected blob has a separate section containing a ranking of 10 ligand predictions and a visualization panel. In the ranking, each ligand prediction is accompanied by a probability score. Higher probabilities mean greater chances of correct predictions. On the other hand, if the probability is low, users should treat the prediction with more caution even if it is at the top of the ranking. Each blob prediction or validation is additionally color-coded to indicate CheckMyBlob's certainty level. Based on the calibration plot of the classification model, validation probabilities above 90% were set to be color-coded with a green icon, between 50–90% with orange, and below 50% with red. Ligand identification color coding uses a 65% unsure threshold instead of 50% to take into account the higher difficulty of this task. To help users navigate the large number of potential ligands, hovering on a ligand's PDB ID shows the compound's full chemical name and diagram. Clicking on a ligand ID opens the ligand's page at the PDB website. In the visualization panel, users can zoom, pan, rotate the view, and hide different parts of the electron density map to analyze the blob. If the set of blobs needs a more in-depth inspection, a script can be downloaded that creates a CheckMyBlob report window in Coot. The report window contains tabs that allow a user to jump to different blobs in the map and display predictions. When the user clicks the button of a possible ligand, Coot performs a simple jiggle fit to fit the ligand into the electron density.

### Case studies

#### Ligand validations

CheckMyBlob was successfully used to recognize ligands in a set of example structures, including the PDB entries:

- 1OGV, 3MB5 and 4IUN, which represent medium and large moieties that were examined in previous ligand prediction studies (9,15);
- 5N0H, which illustrates the recognition of buffer components;
- 2PDT, 1FPX, 4RK3 and 1KWN, which showcase correct predictions of ligands that were misidentified in the original PDB deposits.

The last four of the above-mentioned structures were cases where CheckMyBlob identified the ligand correctly, but the original authors of the PDB deposit either mislabeled a molecule or modeled it incorrectly. In 2PDT, flavin-adenine dinucleotide was reinterpreted by CheckMyBlob as flavin mononucleotide. In 1KWN, the modeled atoms of the ligand had the configuration of l(+)-tartaric acid (TLA) as identified by CheckMyBlob, whereas the deposition authors labeled this moiety (inconsistently with the configuration of the ligand) as d(–)-tartaric acid (TAR). In 1FPX, the modeled *S*-adenosyl-l-methionine (SAM) was reinterpreted as *S*-adenosyl-l-homocysteine (SAH). In 4RK3, disordered glycerol was reinterpreted as a TRIS buffer. The above cases can be viewed as interactive visualizations in Molstack (26,27) at http://molstack.bioreproducibility.org/collection/view/YskIjr2eiLoQelKrwnIG/. The authors of these four deposits (2PDT, 1FPX, 4RK3, 1KWN) agreed with the CheckMyBlob reinterpretation, and the structures were corrected, re-refined, and re-deposited jointly with the original authors into the PDB. In some cases, the improvements of model quality were as large as a 5% reduction of *R*_free_ or a drop of the clashscore by 20 points. This demonstrates that CheckMyBlob can correctly identify ligands, even in sub-optimally modeled structures. Details of this case study can be found in (16).

#### Missed polypeptide and ligands masked by water

The CheckMyBlob web server attempts to recognize not only unmodeled single-molecule ligands but also polymers, i.e., ligands that the PDB encodes as several monomers, and ligands ‘masked’ by water molecules. Figure 2 presents such a case for the validation of PDB deposit 6SYH. CheckMyBlob shows that the deposition authors improperly modeled part of the protein main-chain and did not model two molecules of the crystallization buffer components—HEPES or MES and sulphate or phosphate (the exact nature of the buffer molecules is not possible to determine due to the lack of metadata for this deposit). The significance of this CheckMyBlob use case is twofold. First, our validation procedure is sensitive to some classes of macromolecule modeling errors. In this case, the part of the protein's main-chain (B289-B293) was assigned a wrong sequence and effectively was not bonded to the rest of the protein. Second, when the user chooses to remove waters before the analysis, CheckMyBlob can determine the nature of the ligand even if the density was previously ‘masked’ by the addition of waters in place of the ligand. Both of these types of errors happen frequently during the automatic stages of structure modeling and this example demonstrates the benefit of using the CheckMyBlob server as an aide for crystallographers during the early modeling phase. To facilitate actionable analysis of the server's predictions, CheckMyBlob generates a script for Coot, which lets users jump between blobs and quickly test different ligand fits.

#### Analysis of metal ion predictions

We have also validated the predictive performance of CheckMyBlob on metal ions. Since a significant fraction of structures in the PDB contains poorly modeled metal binding sites (28), we have focused on a high-quality subset of metal ligands. The subset was created by selecting metals that were favorably validated by the CheckMyMetal (CMM) web server (28,29). A metal ion in a structure was considered valid if all CMM validation criteria could be calculated and none of them were reported as an outlier. This procedure resulted in a dataset of 34 932 metal ion ligands, which were passed through CheckMyBlob's validation procedure. The overall classification accuracy on this dataset was 92.3%, although with varying precision and recall for different metal groups (Table 2). The best performance was observed for Zn-like metals (Zn, Mn, Cu, Fe, Ni, Co, Cr, Ga, Ti, V), which are also the most populous metal ligand group. On the other hand, CheckMyBlob was not able to recognize any of the Sr-like metals (Sr, Rb). It is worth noting that CheckMyBlob was not specifically trained to predict metal ions. The prediction of metals could be improved in the future by training a classifier on groups of metals with similar conformations and using additional knowledge about the expected properties of the binding pocket.

**Table 2.** CheckMyBlob's predictive performance on metal ions validated by CMM (28,29). Total number of ligands: 34 932. Total classification accuracy on this dataset: 92.3%

Ligand group	Precision (%)	Recall (%)	F1-score (%)	Ligand instances
MG-like	88.8	87.4	88.1	7 063
CA-like	89.9	93.0	91.4	12 682
ZN-like	96.4	95.2	95.8	14 686
SR-like	0.0	0.0	0.0	19
CD-like	85.1	59.6	70.1	441
HG-like	40.5	41.5	41.0	41

MG-like: Mg, Na, Al; CA-like: Ca, K; ZN-like: Zn, Mn, Cu, Fe, Ni, Co, Cr, Ga, Ti, V; SR-like: Sr, Rb; CD-like: Cd, Ag, Mo, Ru, Pd, Y, Rh, Zr, In; HG-like: all metals with atomic number 55 (Cs) or higher.

### Limitations

The performance of machine learning systems relies on the number and quality of training examples. Indeed, CheckMyBlob requires training data in the form of previous observations of any particular target ligand. Therefore, to detect ligands without examples in the PDB, one can only predict moieties that are structurally similar to the target ligand. Moreover, even though the ligand instances used for training were selected based on several quality criteria, it is impossible to eliminate all noisy examples without removing most of the training data. X-ray electron density maps are noisy by nature and, as evidenced by the case studies, PDB deposits can contain mislabeled ligands. Nevertheless, as the number and quality of PDB deposits grow, it will be possible to tighten the ligand selection criteria for training data even further.

It must also be noted that each prediction should be analyzed on a case-by-case basis. CheckMyBlob's prediction is based solely on the electron density map, and the knowledge about all ligands that might be present in the crystal should be used to select the most probable suggestions, even if they are not at the top of the prediction list. One should consider crystallization conditions, protein buffer components, compounds potentially retained during protein purification, and any potential chemical reactions in between. Moreover, as our study discussing the classification model has shown (16), higher resolution structures have, on average, higher chances of yielding correct predictions.

The current version of the server also has a technical limitation of not being able to fully automatically process models that contain residues that are not present in REFMAC’s standard monomer dictionary. Both ligand validation and identification require a REFMAC run to standardize the map. If the analyzed model contains non-standard or unconventionally named residues, the re-refinement step of the processing pipeline can fail. Currently, the PDB does not host restraint libraries used during refinement; therefore, this might be a problem even during the validation of some PDB deposits. To alleviate this issue, future versions of the server will use automatically generated restraints for moieties that are not present in the standard monomer dictionary.

## DISCUSSION

Here, we have presented an intuitive, easy-to-use web server that aids users in predicting small-molecule ligands in unmodeled fragments of electron density maps and helps validate ligands in existing models. The CheckMyBlob web server is free and open to all users, and there is no login requirement. This work builds on previous efforts to predict ligands using machine learning algorithms (16) and extends that approach by clustering ligands into groups and by taking into account water molecules and metal ions. In case studies, we have shown how CheckMyBlob can be used to find errors in PDB deposits and identify missing ligands. We feel that this work is a valuable contribution to the scientific community by providing a way of ligand validation that is complementary to density fit metrics. Moreover, CheckMyBlob is complementary to structure-remediation servers, such as PDB_REDO (30), which do not handle ligand misinterpretation. Finally, CheckMyBlob can help find ligands that were unmodeled or masked by water molecules. By providing a method for autonomous detection of blobs, the presented method has the potential of being another step toward fully automated model building.

## DATA AVAILABILITY

The ligand clustering code and prediction evaluation scripts are available at GitHub (https://github.com/dabrze/cmb_server_validation). The ligand datasets used for evaluating CheckMyBlob are hosted at bioreproducibility.org (https://bioreproducibility.org/test-data-checkmyblob-server) and Zenodo (10.5281/zenodo.4554473).
